# Male Breast Cancer: An Updated Review of Epidemiology, Clinicopathology, and Treatment

**DOI:** 10.1155/2022/1734049

**Published:** 2022-05-24

**Authors:** Guoliang Zheng, Jose Pablo Leone

**Affiliations:** ^1^Department of Medicine, St Elizabeth Medical Center, A Teaching Hospital of Boston University, 736 Cambridge Street, Boston, MA, USA; ^2^Dana Farber Cancer Institute, 450 Brookline Avenue, Boston, MA, USA

## Abstract

Male breast cancer (MaBC) is a rare clinical entity, which makes up approximately 1% of all breast cancers. However, the incidence of MaBC has been steadily increasing over the past few decades. The risk factors for MaBC include age, black race, family history of breast cancer, genetic mutations, liver cirrhosis, and testicular abnormalities. The majority of patients with MaBC present with painless lumps, and about half of the patients have at least one lymph node involved at the time of diagnosis. The treatment of MaBC models that of female breast cancer (FeBC), but this is mainly due to lack of prospective studies for MaBC patients. The treatment modality includes surgery, adjuvant radiation, endocrine therapy, and chemotherapy. However, there are some distinct features of MaBC, both clinically and molecularly, that may warrant a different clinical approach. Ongoing multinational effort is required, to conduct clinical trials for MaBC, or the inclusion of MaBC patients in FeBC trials, to help clinicians improve care for MaBC patients.

## 1. Introduction

Breast cancer in men is a relatively rare disease and accounts for only 1% of the breast cancer population. As with all other rare diseases, it has been challenging to conduct prospective clinical studies in male breast cancer (MaBC) as evidenced by several prematurely closed clinical trials due to the lack of enrollment. This situation is exacerbated by the exclusion of male participants in female breast cancer (FeBC) clinical trials in the past. Our current knowledge of male breast cancer is largely derived from small retrospective studies, often single-center experience, and as a result, approaches to treating MaBC are extrapolated from guidelines in FeBC management. There is rising evidence that MaBC has distinct clinical features that trace back to molecular levels (i.e., genomics and tumor subtypes), and different treatment approaches may improve morbidity and mortality. In this study, we review the epidemiology and risk factors, highlight the similarity and differences between MaBC and FeBC at molecular level (including genomics and tumor characteristics), characterize clinical features and diagnostic modalities, and summarize treatment regimens and future directions in research.

### 1.1. Epidemiology

MaBC is a rare disease and makes up only approximately 1% of all breast cancers in the United States and worldwide. [1] Similar to female breast cancer, the incidence rate continues to rise [2]. It is estimated that there will be 2620 new cases of male breast cancers diagnosed in the United States in 2020, compared to only 900 cases in 1991 [3]. The age-adjusted incidence rate has increased to 1.32 per 100,000 men in 2017, from 0.90 per 100,000 in 1980 as outlined by the Surveillance, Epidemiology, and End Results (SEER) [4].

### 1.2. Risk Factors

#### 1.2.1. Age and Ethnicity

The incidence of MaBC varies by ethnicity and age, with the highest incidence rate of 1.89/100,000 in non-Hispanic black population, which is significantly higher than that of the non-Hispanic White population (1.3/100,000), the Asian population (0.7/100,000), and the Hispanic population (0.8/100,000) [1]. Higher incidence rates of MaBC are observed in South and Central Africa, which is possibly due to hyperestrogenism in the setting of more prevalent infectious hepatic disease [5]. In the retrospective study by O'Malley et al, non-Hispanic blacks and whites have similarly poor 5-year survival rates (57% and 66%, no confidence interval or *p* value available), which are noticeably worse than that of other race/ethnicities (75%) [6].

The incidence rate increases with age, with a significant increase at Age 50 (showing an incidence rate of 1.7/100,000) and plateauing at Age 80 and above (with an incidence rate of 8.3/100,000) [4]. In a large retrospective cohort study of 19,795 MaBC patients, the median age at diagnosis was 65 years, with 15% of patients diagnosed at age <50 years [7].

#### 1.2.2. Family History

It is estimated from multiple population studies that 15–20% of male patients diagnosed with breast cancer have at least one first degree relative that developed breast cancer [8]. Similar to women, men who have either a female or male first degree relative with breast cancer have a 2- to 3-fold increased risk of developing breast cancer. The risk substantially increases with increasing numbers of affected family members [9]. Although the odds ratio is not quantified, there are case reports of association of MaBC with a family history of both Lynch syndrome and Cowden syndrome [10, 11]. However, screening mammography is not recommended for men with family history of breast cancer, as the absolute risk of MaBC in this population is still considered to be very low.

#### 1.2.3. Klinefelter Syndrome

Klinefelter is a rare genetic disease that occurs when men gain one extra X chromosome, which clinically manifests as gynecomastia and testicular dysgenesis. Men with Klinefelter syndrome carry significantly increased risk (20- to 50-fold more) of MaBC compared with the general male population, and it is hypothesized the increased risk is primarily due to their low androgen and high gonadotrophin state [12].

#### 1.2.4. Hormonal Imbalance

It is interesting to note that meta-analysis pooling results from 10 cohort studies and 11 case-control studies discovered an association of MaBC with increased estrogen level (rather than reduced androgen level) [13]. Similar to women, men with diseases, such as obesity and alcohol dependence that result in pathological increase in endogenous estrogen level, are at elevated risk of developing breast cancer [14, 15]. Two unique risk factors to males are liver cirrhosis and testicular dysfunction (such as undescended testes and congenital inguinal hernia), both of which also increase men's risk of developing breast cancer later in life [16, 17] Gynecomastia is frequently associated with increased estrogen level and has been proposed to be associated with increased risk of MaBC. However, in a series of prospective studies, gynecomastia does not increase the probability of developing MaBC [18–20].

#### 1.2.5. Environmental Exposure

Several occupational hazards, including hot working environments, ionizing and electromagnetic radiation, or exposure to chemical compounds such as combustion products, have been associated with increased risks of developing MaBC [21–23]. However, these findings have been limited to case reports and case series.

### 1.3. Genetics

It is well characterized that mutations of both BRCA1 and BRCA2, tumor suppressor genes involved in DNA repair, are implicated in FeBC. Approximately 10–15% of FeBC are associated with these autosomal dominant mutations, which confer a cumulative 45–65% life time risk [24]. In men, BRCA2 mutations and, less frequently, BRCA1 mutations are established risk factors of breast cancer. In population studies unselected for family history, 4–33% of male breast cancer patients harbor BRCA2 mutations and 0–6% harbor BRCA1 mutations [25–39]. The calculated average BRCA2 mutations from pooled data are 10% (83/840). This broad range is, in part, explained by the differences in sample size. For instance, the study by Csokay et al only includes 18 patients. However, 6/18 patients were found to have BRCA2 mutations [28]. Most of these studies are small retrospective studies in single institutions, and they highlight the importance of multicenter/country collaboration to identify more accurate incidence rates. In two studies that are selected for family history, BRCA2 mutation frequencies ranged from 37 to 40%, and BRCA1 mutations were found in about 5% of study subjects [40, 41].

In a recent retrospective study involving the analysis of multigene panel testing of 708 MaBC patients, 97 patients (13.7%) have at least one pathogenic variant in breast cancer susceptibility gene, 11% tested positive for BRCA2, 4.1% for CHEK2, 1.5% for ATM, 1.3% for BRCA1, 0.6% for NF1, and 0.5% for PALB2 [42]. Similar results are observed in another retrospective study [43]. Mutations of CHEK2, a cell cycle check point kinase also involved in DNA repair, have been implicated in the literature as a risk factor for MaBC and, in particular, CHEK2 1100delC with as high as 10-fold increased risk [44]. However, there are also conflicting findings from other studies, where researchers did not find any association between CHEK2 and male breast cancer [45–47]. Another frequently discussed gene mutation is PALB2, which involves encoding a protein in the BRCA2-related pathway. The reported frequency ranges from 0.8 to 6.4% in MaBC patients [39, 48–50]. The risk of developing MaBC in patients with PALB2 mutation is 4- to 6.6-fold higher than those not carrying the PALB2 mutations [42, 50]. Other implicated genes in the literature also included CYP17, RAD51B, and PTEN mutations associated with Cowden syndrome [51–53]. Patients carrying these gene mutations, in particular, BRCA2, are clearly at risk of developing MaBC. The National Comprehensive Cancer Network (NCCN) recommends that these patients self-breast examination and clinical breast examinations should be carried out for MaBC screening twice each year. However, recommendations for annual mammography are unclear [54]. Gene mutations implicated in MaBC are listed in Table 1.

### 1.4. Clinical Features and Diagnostic Imaging

Similar to FeBC patients, the majority of MaBC patients (approximately 75%) present with a painless lump, most frequently in the retro-areolar area. Most patients have early nipple involvement, including retraction, discharge, or ulceration, as the rudimentary breast ducts are located directly beneath the nipple [55–58]. Because of the rarity of MaBC and the lack of established screening guidelines in men, there is often a delay in diagnosis, with one study reporting a mean duration of 21 months since the onset of symptoms [59]. For this reason, approximately 46.7% of men have disease involving at least one lymph node at the time of diagnosis, as reported in a recent study carried out by the International Male Breast Cancer Program (IMBCP) [60]. Often times, patients can present with gynecomastia, making it difficult to distinguish from MaBC. Therefore, in these cases, imaging is required for further evaluation. The American College of Radiology recommends that men younger than 25 years with a palpable mass should undergo bilateral ultrasound evaluation, and men older than 25 years should undergo bilateral mammography; however, if the mammography is indeterminate, ultrasound should be performed as an adjunct tool [61]. Most common findings on the ultrasound are irregular, hypoechoic retro-areolar masses that appear spiculated and have variable vascularity, along with mammography often showing similar spiculated and radio-dense irregular retro-areolar masses [62]. Similar to its use in diagnosis of FeBC, mammograms have a high sensitivity of 92–100% and high specificity of 90–96% in diagnosing MaBC [63]. However, there is currently no evidence that support screening for asymptomatic men.

### 1.5. Tumor Characteristics and Biology

The most prevalent MaBC is invasive ductal carcinoma, and because of anatomical differences (as male breasts consist of ducts without terminal lobules), the prevalence of invasive lobular carcinoma is much lower in males (1–2%) than in females (15%) [64]. The largest multicenter study of MaBC to date conducted by IMBCP, involved review of 1483 breast tissues, displayed a similar prevalence: 85% of invasive ductal carcinoma, 1.4% of invasive lobular carcinoma, and the remaining comprised of mixed ductal and lobular carcinoma (5.9%), papillary (3%), and mucinous (1.9%) [65]. Approximately 50% of these invasive cancers are histological grade 2, 22% are grade 1, and 27% are grade 3 [65]. Only about 10% of MaBC patients presented with a precursor lesion compared to 15–30% of women, which is possibly due to the rare manifestation of ductal carcinoma in situ as palpable masses and the lack of image-based screening in men [66].

As for tumor subtypes, current available data are derived from retrospective studies. The study by IMBCP examined 1483 patients diagnosed between 2001 and 2010, which discovered that 99.3% of these patients were ER positive, 81.9% PR positive, 96.9% AR positive, and 8.7% HER2 positive. The Ki67, a marker of cell proliferation, had <20% positive cells in 75% of the samples assessed, and the other 25% had >20% positive cells with a high Ki-67 expression [60]. The molecular subtyping of breast cancer can be further subdivided, according to the luminal classification, into Luminal A (ER+, PR+, HER2-, and low Ki67), Luminal B-like HER2 negative (ER+, HER2-, and high Ki67 or PR-), Luminal B-like HER2 positive (ER+, HER2+, any Ki-67, or any PR), nonluminal (HER2+, ER-, and PR-), and basal (triple negative). In this study, 42% were luminal A, 49% Luminal B-like/HER2-, 9% HER2+, and 0.3% triple negative [60]. The results of this large international study were consistent with those derived from SEER, showing that MaBC is predominantly ER/PR positive and HER2 negative. Interestingly, ER is known to have alpha and beta fractions, and the two fractions reside in different tissues of the human body-alpha is often found in endometrium and ovaries, whereas beta is often found in kidney, brain, bones, and prostate [67]. In contrast to FeBC, which contains mostly ER-alpha receptors, the majority of MaBC have ER-beta receptors, indicating possible different biological activities at the molecular level between MaBC and FeBC. This may warrant a different approach to MaBC in terms of anti-estrogen therapy compared with FeBC [68].

### 1.6. Prognosis

The prognosis for MaBC is generally worse than that of FeBC. The study by Wang et al investigating 16025 male and 1,800,708 female patients diagnosed with breast cancer from the National Cancer Data Base (NCDB) between 2004 and 2014 demonstrated that overall mortality and mortality at three and five years are higher in men when compared to women even after adjusting for clinical characteristics, treatment factors, age, race/ethnicities, and access to care [69]. In a study carried by Leone et al. examining 2992 MaBC patients from SEER between 2003 and 2012, age at diagnosis, tumor grade, stage, surgery, radiotherapy, ER, and marital status have been identified to carry prognostic value in MaBC [70]. Importantly, tumor subtypes have been shown to have clear impact on prognosis in two additional studies looking at the SEER OS data of 960 MaBC patients from 2010 to 2012 and 1187 MaBC patients from 2010 to 2013. These two studies show that patients with HER2+ and triple-negative (TN) tumor subtypes have significantly worse prognoses [71, 72].

### 1.7. Early-Stage MaBC Treatment

**Figure 1:**
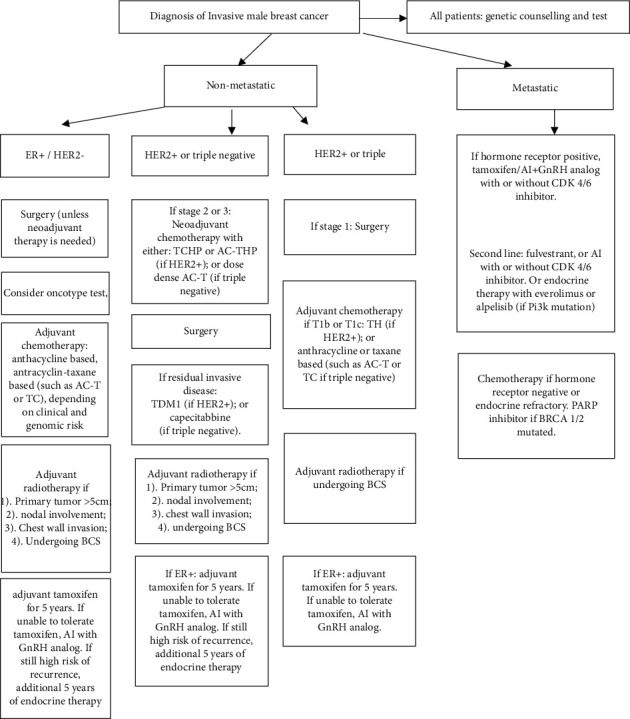
Treatment algorithm for male breast cancer.

Given the relative rarity of MaBC (as compared to FeBC), most of the treatment modalities in MaBC are extrapolated from the current standard of care of FeBC. Although there are similarities in disease characteristics, MaBC has distinct features that warrant a specific clinical approach. A treatment algorithm is illustrated in Figure 1.

Similar to FeBC, for early-stage disease in men, surgery plays a fundamental role. However, unlike FeBC, in which 2/3 of patients undergo breast conserving surgery (BCS) and 1/3 mastectomy, the majority of MaBC patients undergo mastectomy, leaving a small percentage of men (10–24%) who are treated with BCS. Even in MaBC patients with T1N0 disease, only 18% of patients underwent BCS [73–75]. This observation could be, in part, due to the central location of most MaBC, which can involve the nipple-areolar complex. Furthermore, MaBC patients may prefer mastectomy to avoid breast irradiation altogether, contributing to a small trend of decreasing BCS between 2004 and 2014 observed by Yadav et al. [76]. In regard to prognosis, several retrospective studies find similar overall survival rates in MaBC patients who either underwent mastectomy or BCS [73–78]. If BCS is desired in MaBC patients with large tumors or nodal involvement, neoadjuvant therapy (endocrine or cytotoxic) can potentially be employed to decrease tumor size so that BCS can be feasible. This, however, is only used in limited cases [79].

Axially lymph node dissection (ALND) has been a standard surgical procedure in MaBC patients, but is associated with significant morbidity, including lymphedema, infection, and axillary paresthesia. Sentinel lymph node biopsy (SLNB) is underutilized in MaBC patients when compared to FeBC patients, even though several case series has shown similar accuracy in predicting axillary nodal status [80–83].

In FeBC, adjuvant radiotherapy is recommended for patients with metastasis to four or more lymph nodes, any involvement of internal mammary or supraclavicular nodes, invasion of chest wall after mastectomy, and for those who undergo BCS [84]. Recommendations for FeBC patients with T1-T2 tumors with involvement of 1–3 lymph nodes require more clinical judgement, especially for the subset of patients with decreased risk of recurrence. Because there is lack of prospective data on radiotherapy in MaBC, the recommendations for FeBC also apply to MaBC, to reduce locoregional failure, disease recurrence, and breast cancer mortality. In a recent study analyzing SEER data between 1998 and 2003, there was improved OS for case-matched patients who underwent postmastectomy radiotherapy (PMRT) (83% vs 54%, *p* < 0.001) [85]. Subgroup analysis in the same study also demonstrated improved OS in those with 1–3 lymph nodes (79% vs 72%, *p* < 0.05) as well as 4+ lymph nodes involved (73% vs 53%, *p* < 0.001). In another meta-analysis involving 29 studies and 10,965 MaBC patients after mastectomy, PMRT also demonstrated improved locoregional control and survival [86]. However, the utilization of PMRT varies greatly from 2 to 100%, with a mean of 64%, and it generally appears underutilized in MaBC patients [86]. Radiotherapy, as part of the standard therapy for MaBC patients who underwent BCS, is also not administered systematically. In one study, only 35.4% of patients received radiotherapy after lumpectomy, and in another study, about 42% received radiotherapy [74, 75]. The reasons for the underutilization of adjuvant radiotherapy in MaBC patients remain unclear. However, there is a trend of increasing utilization of PMRT (50% in 2004 and 52% in 2014) and radiation therapy after BCS (66% in 2004 to 74.6% in 2014) as identified by Yadav et al. [76]. In recent years, innovative techniques such as hypo-fractionated regimen or controlled regional delivery via computed tomography scan planning are being developed to improve precision and minimize heart and lung toxicity in FeBC [87, 88]. However, these techniques are yet to be examined in MaBC.

The use of adjuvant chemotherapy-anthracycline-based, anthracycline-taxane-based, and cyclophosphamide, methotrexate, and 5-fluorouracil (CMF) has been associated with improved OS in stage II and III disease [60, 76]. A phase 2 clinical trial, evaluating CMF in 31 men with node-positive breast cancer, has reported 20-year results. The study concluded that patients had significantly better OS (80% OS at 5 years, and 42% at 20 years) compared to historic rates [89]. Another retrospective study found reduced recurrence and improved OS in stage II MaBC patients with nodal involvement receiving anthracycline-based regimens [90]. Currently, MaBC patients receiving adjuvant chemotherapy tend to have larger tumors, lymph node involvement, hormone receptor-negative disease, and younger age at diagnosis [53]. In hormone receptor-positive FeBC, the 21 gene recurrence score has been used for prognosis and to predict benefit from adjuvant chemotherapy. As compared with FeBC, MaBC is more likely to have scores ≥31 (12.4% vs 7.4%) and also more likely to have scores <11 (33.8% vs 22.1%) [91]. The recurrence score is prognostic in both men and women [92].

Since over 90% of MaBC patients have hormone receptor positive disease, endocrine therapy is an important part of MaBC treatment. Tamoxifen has, so far, been the most widely used anti-estrogen therapy in both FeBC and MaBC. There are no prospective studies evaluating the efficacy of tamoxifen in MaBC. However, there are several retrospective studies showing improved OS with adjuvant tamoxifen use in early-stage MaBC, especially with node-positive disease [93, 94]. Compliance to tamoxifen in MaBC has only been investigated in a few studies. One study found 65% of men reported taking the tamoxifen after 1 year, 46% after the 2^nd^ year, 29% after the 3^rd^ year, 26% after the 4^th^ year, and 18% after the 5^th^ year [95]. The reduced compliance to tamoxifen may be secondary to the side effect profile, including hot flashes, sexual dysfunction, reduced libido, mood lability, and venous thromboembolism [96, 97]. Another commonly used endocrine therapy in postmenopausal women is an aromatase inhibitor (AI). In a matched cohort study comparing 5-year OS of FeBC and MaBC after AI, women have a significantly better OS than men (85% vs 73% *p*=0.028) [95]. In men, 80% of estrogen is produced by peripheral conversion of androgen via aromatase and the other 20% directly secreted by the testicles. AI work by inhibiting peripheral conversion of androgen to estrogen. However, AI as a monotherapy may not be as effective in MaBC, because the reduced serum estrogen level activates negative feedback loop to a functioning hypothalamus to secrete luteinizing hormone (LH) and follicle-stimulating hormone (FSH). Therefore, the testicles secret additional testosterone as a substrate for estrogen production [98]. In addition, testicles secret estrogen that accounts for about 20% of the total estrogen level. This is supported by studies, showing that estradiol level is suppressed to 14.1 pg per milliliter after anastrozole in healthy men vs less than 1 pg per milliliter in postmenopausal women [99, 100]. Therefore, for AI to achieve its desired effect, it would require either surgical or chemical castration so that its use will not lead to increased testosterone production. A GnRH analog can be added to AI therapy to obliterate the secretion of LH and FSH in those who cannot tolerate tamoxifen. This has been recommended by several guidelines, including NCCN, and American Society of Clinical Oncology (ASCO) [101, 102]. However, the addition of GnRH to AI compared to AI alone in patients with MaBC showed only marginal improvement in progression-free survival (PFS) and OS in two retrospective studies (1st study: the median PFS was equal to 10.0 months, while the median OS was equal to 39.0 months and 2nd study: 1.6 months vs 6 months for PFS and 29.7 months vs 22 months for OS; both *P*=.05) [103, 104]. In the MALE trial, 56 hormone receptor-positive MaBC patients were randomized to receive tamoxifen, tamoxifen with GnRH, and AI with GnRH—no single arm received only AI. The study showed a consistent decrease in estradiol levels in the two combination arms, but no survival data were reported [105]. The coadministration of GnRH and AI is associated with significant sexual dysfunctions as it results in both the reduction of testosterone and estrogen. To summarize, there is consistent reduction of estradiol levels observed in both retrospective and prospective studies, but whether GnRH plus AI is superior to AI monotherapy still needs to be evaluated in prospective studies. Side effect profiles should be taken into consideration when prescribing GnRH in addition to AI.

Finally, in a very small subset of MaBC patients who are HER2 positive, mirroring treatment from FeBC is recommended with HER2-directed therapy, as there are no prospective studies that evaluate the efficacy of its use in MaBC.

### 1.8. Metastatic MaBC Treatment

Similar to treatment algorithm in FeBC, endocrine therapy has been proposed as a first-line treatment in hormone receptor-positive MaBC. Tamoxifen is a preferred first-line agent for MaBC. However, in patients whose disease progress while on tamoxifen treatment, AI in combination with GnRH analog should be used rather than AI alone, as discussed above. A retrospective study of 19 metastatic MaBC patients treated with AI and GNRH showed that 36.8% of patients achieved partial response, 36.8% achieved stable disease, 15.8% with progressive disease, and median PFS and OS of 12.5 months and 35.8 months, respectively [106]. Three out of 4 patients had improved response with the combination therapy, having previously been on AI monotherapy. Once the disease becomes refractory to both tamoxifen and AI with GnRH analog, fulvestrant can be considered, although its use in MaBC has not been well studied. According to one pooled analysis of 23 metastatic MaBC patients, 40% received it as first- or second-line treatment with the remaining as third line or beyond. The study concluded that 26.1% of patients achieved partial response and 47.8% of patients achieved stable disease, suggesting the potential role of fulvestrant in treating metastatic MaBC [107].

The use of CDK 4/6 inhibitors in combination with ET has doubled the PFS in women with HR+/HER2-metastatic disease, compared to those using ET alone [108]. A retrospective study investigating the use of CDK 4/6 inhibitor palbociclib (PAL) along with ET in metastatic MaBC between 2015 and 2017 showed that the mean duration of therapy is longer in the PAL group than in the non-PAL group. The maximum response rate (complete response and partial response) is higher in the PAL + ET group compared with the ET alone group (33.3% vs 12.5%) [109]. The use of real-world evidence, as conducted in this particular study, was very helpful, given that the trials that led to the approval of palbociclib for metastatic breast cancer excluded men.

The use of everolimus in combination with AI has also been demonstrated to improve median PFS, compared to administering AI alone (6.9 vs 2.8 months *p* < 0.001) in advanced FeBC that is HR+/HER2- [110]; however, in MaBC, its use has not been evaluated in prospective studies or large case series, but two case reports have described good response in men to the combination of everolimus with either exemestane or tamoxifen [111, 112].

Chemotherapy in advanced MaBC is primarily used in patients with hormone receptor-negative tumors, disease that becomes resistant to ET, or with visceral crisis that requires treatment with swift response [113]. One retrospective study evaluating 50 metastatic MaBC patients previously treated with ET compared anthracycline-based regimen and anthracycline-free regimen, and found no significant difference in PFS and OS [114]. In FeBC patients, it is concluded that single-agent chemotherapy has similar efficacy to multiagent chemotherapy with less toxicity [115]. There is one case series evaluating the use of single-agent eribulin in 23 MaBC. Patients received a median of 6 cycles, and nearly half of the patients achieved clinical responses [116].

A substantial percentage of both FeBC and MaBC express AR. Naturally, therapies targeting AR have been proposed as potential treatments. Although AR has an oncogenic role in prostate CA and AR antagonizing strategies with anti-androgenic drugs are effective, the role of AR in BC is unclear. Some clinical studies investigating anti-androgenic therapies, including two retrospective studies and 1 case report, have looked at CYP17A1 inhibitor cyproterone acetate with or without GnRH analog, and showed an overall 53% response rate. The two recent clinical trials show no significantly improved PFS in metastatic HR^+^/HER2^−^ FeBC patients with the addition of enzalutamide to endocrine therapies [117–121]. More recently, there has been an increasing interest in AR agonist therapy after Hickey et al. undertook a large-scale study showing how AR can act as a tumor suppressor rather than driver in ER + BC, by opposing ER transcriptional activity [122]. A recent randomized phase 2 study examined the use of a nonsteroidal tissue-selective AR modulator—enobosarm—in heavily pretreated metastatic ER + FeBC, which displayed a clinical benefit rate at 24 weeks, and an objective response rate at approximately 30% in both 9 mg and 18 mg groups. This medication was well tolerated [123]. As MaBC is almost universally ER+/AR+, AR agonist therapy should be further investigated.

Given the frequency of BRCA alterations in MaBC, poly-ADP-ribose-polymerase (PARP) inhibitors can be a relevant treatment option. Its use has been studied in two phase III trials: OLYMPIAD and EMBRACA. In OLYMPIAD, a total of 295 patients (7 of which were MaBC patients with BRCA-mutated HER2-negative metastatic disease) were enrolled to receive either an oral PARP inhibitor olaparib or a physician's choice single-agent chemotherapy (TPC). There was no subgroup analysis for the MaBC patients, but PFS was significantly longer in the olaparib arm (7.0 vs 4.2 months *p* < 0.001), while olaparib had a better response rate (59.9% vs 28.8% *p* < 0.001) with a better toxicity profile than TPC [124]. In the EMBRACA trial, a total of 431 patients (9 of which were male patients with advanced breast cancer and germline BRCA mutations) were enrolled to receive either talazoparib or single-agent chemotherapy. PFS was significantly longer in the talazoparib arm (8.6 vs 5.6 months; *p* < 0.001), which also showed superior patient-reported outcome [125].

## 2. Conclusion

MaBC is a relatively rare disease with increasing incidence, yet it is understudied with most current clinical approaches extrapolated from data in FeBC. From the available data, we can conclude that MaBC has distinct molecular and clinicopathological features that may warrant different clinical approaches from FeBC. Various novel therapeutics, including PARP inhibitors and anti-androgen therapies that are undergoing investigations in FeBC, may also be successful in MaBC. We are already seeing a trend of clinical trials that now include MaBC patients to provide evidence base that will inform future treatment in MaBC. Hopefully, collective multinational effort will also facilitate the conduction of exclusively MaBC prospective trials in the near future.

## Figures and Tables

**Table 1:** Gene mutations implicated in MaBC.

Genes implicated	Frequency
BRCA2	11%^42^
BRCA1	1.30%^42^
CHEK2	4.10%^42^
PALB2	0.8–6.4%^48-51^
ATM	1.50%^42^
NF1	0.60%^42^

## Data Availability

The data can be obtained from the corresponding author upon request.

## Abbreviations

AI: Aromatase inhibitor
GnRH: Gonadotropin-releasing hormone
PARP: Poly-ADP-ribose-polymerase
BCS: Breast conserving surgery
TCHP: Docetaxel, carboplatin, trastuzumab, and pertuzumab
AC-THP: Adriamycin, cyclophosphamide, trastuzumab, and pertuzumab
TDM1: Trastuzumab.
